# ANGPTL8/Betatrophin R59W variant is associated with higher glucose level in non-diabetic Arabs living in Kuwaits

**DOI:** 10.1186/s12944-016-0195-6

**Published:** 2016-02-11

**Authors:** Mohamed Abu-Farha, Motasem Melhem, Jehad Abubaker, Kazem Behbehani, Osama Alsmadi, Naser Elkum

**Affiliations:** Biochemistry and Molecular Biology Unit, Dasman Diabetes Institute, Kuwait City, Kuwait; Dasman Genome Center, Dasman Diabetes Institute, Kuwait City, Kuwait; Sidra Medical and Research Center, Doha, Qatar

**Keywords:** ANGPTL8, LDL, HDL, Lipid metabolism, Glucose metabolism, Sanger sequencing, Single nucleotide polymorphism

## Abstract

**Background:**

ANGPTL8 (betatrophin) has been recently identified as a regulator of lipid metabolism through its interaction with ANGPTL3. A sequence variant in ANGPTL8 has been shown to associate with lower level of Low Density Lipoprotein (LDL) and High Density Lipoprotein (HDL). The objective of this study is to identify sequence variants in ANGPTL8 gene in Arabs and investigate their association with ANGPTL8 plasma level and clinical parameters.

**Methods:**

A cross sectional study was designed to examine the level of ANGPTL8 in 283 non-diabetic Arabs, and to identify its sequence variants using Sanger sequencing and their association with various clinical parameters.

**Results:**

Using Sanger sequencing, we sequenced the full ANGPTL8 gene in 283 Arabs identifying two single nucleotide polymorphisms (SNPs) Rs.892066 and Rs.2278426 in the coding region. Our data shows for the first time that Arabs with the heterozygote form of (c.194C > T Rs.2278426) had higher level of Fasting Blood Glucose (FBG) compared to the CC homozygotes. LDL and HDL level in these subjects did not show significant difference between the two subgroups. Circulation level of ANGPTL8 did not vary between the two forms. No significant changes were observed between the various forms of Rs.892066 variant and FBG, LDL or HDL.

**Conclusion:**

Our data shows for the first time that heterozygote form of ANGPTL8 Rs.2278426 variant was associated with higher FBG level in Arabs highlighting the importance of these variants in controlling the function of betatrophin.

## Background

Diabetes prevalence is reaching epidemic proportion with increasingly more people affected by either Type 1 diabetes (T1D) that is caused by insulin deficiency or Type 2 Diabetes (T2D) that is caused by insulin resistance [1–6]. Regeneration of beta cells in both diseases has been regarded as an ultimate goal that could improve or replace diabetes therapies for both diseases [7]. Accumulated research data has shown that beta cells have the capacity to compensate for increased insulin demand under physiological conditions such as pregnancy as well as insulin resistance in pathological conditions such as obesity [8]. Factors such as the gut derived hormones glucagon like peptide 1 (GLP1) and glucose-dependent insulin-tropic polypeptide has been shown to increase insulin secretion and to increase beta cell proliferation [9].

ANGPTL8 also called betatrophin has been recently shown to affect beta-cell proliferation and suggested as a possible target for beta-cell regeneration [9, 10]. Earlier studies gave betatrophin the name ANGPTL8 protein due to its sequence similarity to members of the angiopoietin like protein (ANGPTL) family [11, 12]. It has been shown to interact with ANGPTL3 and regulate triglyceride (TG) and fatty acid metabolism. Ren et al. showed that ANGPTL8 was induced during adipogenesis of primary mouse and human adipocytes as well as 3T3 L1 adipogenesis [13]. Reduction in betatrophin was also associated with reduced adipogenesis which was distinguished by reduced TG [13]. Similarly, mice lacking betatrophin had a similar TG level in the fasting state compared to wild type and a rather lower TG level after feeding [13]. They also failed to properly store fatty acids in adipose tissue and showed a slower weight gain compared to wild type littermates [13]. Nonetheless, they did not show any changes in glucose homeostasis in mice fed with chow or high fat diet. In humans, it has been shown that betatrophin was increased in T1D [14] and T2D subjects [15–20].

A coding variant in ANGPTL8 (Rs2278426, c.194C > T) responsible for an amino acid change in the encoded protein (R59W) has been shown to be associated with lower plasma LDL and HDL in African American and Hispanic in the Dallas Heart Study [12]. Ethnic variation has been observed in the minor allele frequency (MAF) of this variant and its association with LDL and HDL, as Europeans had lower MAF and did not show any association between R59W and levels of LDL and HDL in the Dallas Heart Study [12]. To study the effect of ANGPTL8 sequence variants in Arabs, we used Sanger sequencing to identify novel ANGPTL8 variants. We used Arab subjects from our sample cohort to identify sequence variations in ANGPTL8 and to study their association with the level of circulating ANGPTL8 and other metabolic risk factors particularly FBG, TG, LDL and HDL.

## Research design and methods

### Study participants

This is a cross sectional survey undertaken on 283 adult (>18 years old) Arabs living in Kuwait. As previously described, subjects included in this study were selected randomly from a large cohort that has been randomly collected from multi-ethnic subjects living in Kuwait [17, 21–24]. Samples were collected from the six governorates of the state of Kuwait, where random sample was collected from each stratum with proportional allocations. The study conformed to the principles outlined in the Declaration of Helsinki and was approved by the Scientific Advisory Board and Ethical Review Committee at Dasman Diabetes Institute (DDI). An informed written consent was obtained from all the participants before their enrolment in the study. Subjects with Diabetes or CVD and taking any medications were excluded from the study.

### Anthropometric and physical measurements

Physical and anthropometric measurements included body weight, height, waist circumference (WC) and Blood Pressure (BP). As previously described [17, 21, 23, 24], height and weight were measured, with participants wearing light indoor clothing and barefooted, using calibrated portable electronic weighing scales and portable inflexible height measuring bars. WC was measured using constant tension tape at the end of a normal exhalation, with arms relaxed at the sides, at the highest point of the iliac crest and at the mid-axillary line. BP was measured with Omron HEM-907XL Digital sphygmomanometer. The average of 3 BP readings, with 5 to 10 min rest between each, was obtained. BMI was calculated using the standard BMI formula: body weight (in kilograms) divided by height (in meters squared).

### Laboratory measurements

Blood samples were obtained after fasting overnight for at least 10 h and analyzed for FBG, HbA1c, fasting insulin, and lipid profiles that included TG, TC, LDL and HDL. Glucose and lipid profiles were measured on the Siemens Dimension RXL chemistry analyzer (Diamond Diagnostics, Holliston, MA). HbA1c was determined using the VariantTM device (BioRad, Hercules, CA). All laboratory tests were performed by certified technicians at the clinical laboratories of DDI using the Ministry of Health approved methods and quality standards.

### ELISA betatrophin level

To measure metabolic markers, blood was drawn into EDTA tubes. Plasma was obtained after centrifugation, aliquoted and then stored at −80 °C. Betatrophin concentration was determined using ELISA (Wuhan EIAAB Science co) as described previously [14, 15]. A range of betatrophin concentrations were used to spike in plasma at different dilution factors. The assay showed linearity at dilutions ranging from 1:10–1:40. Recovery of the known proteins ranged from 85 to 109 %. No significant cross reactivity with other proteins has been observed. Intra-assay coefficients of variation were 1.2 to 3.8 %, while the inter-assay coefficients of variation were 6.8 to 10.2 %.

### DNA sequencing

**Fig. 1:**
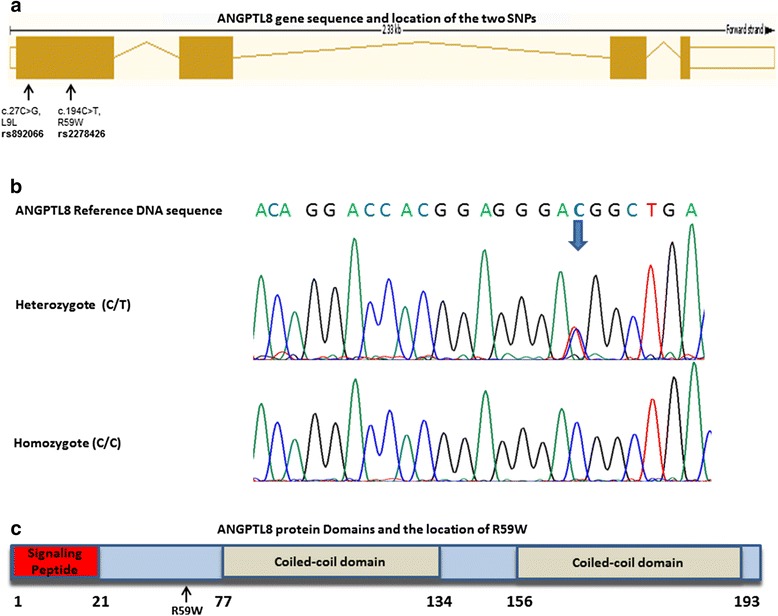
ANGPTL8 gene and protein sequence. **a** A diagram showing the sequence location of various introns and exons of the ANGPTL8 gene and the location of the sequence variants. **b** Sanger sequencing raw data showing the location of the heterozygote and the Homozygote genotype nucleic acid bases in respect to the ANGPTL8 reference sequence. **c** A diagram showing the location of the R59W variant in respect to ANGPTL8 protein domains

Genomic DNA was extracted from whole blood which was withdrawn from different study participants as outlined above. DNA was extracted from the blood using Gentra Puregene kit (Qiagen) as per the manufacturer’s protocol. Coding exons and exon-intron boundaries (splice sites) of the TD26 gene were amplified using genomic DNA (25 ng per reaction) via polymerase chain reaction (PCR), in 25 μl amplification mix containing the corresponding M13 sequence-tagged primers pair and GoTaq® Green Master mix (Promega). Amplified PCR products were purified using ExoSAP-IT (Affymetrix) for the subsequent Sanger’s sequencing, using Big Dye terminator cycle sequencing kit (Applied Biosystems). Bi-directional sequencing reactions were carried out on each purified product using the generic M13 primer sequence tags. The sequencing products were then cleaned to remove unincorporated dye using DyeEx 2.0 Spin Kit (Qiagen), and finally denatured and loaded on the ABI Prism 3730xl Genetic Analyzer (Applied Biosystems) for sequencing. The resulting sequence contigs were analyzed and aligned against a reference sequence using the Chromas Pro software to detect the sequence variations. Two SNPs were identified from the analysis (c.27C > G, L9L and c.194C > T, A59W) Fig. 1a, b & c.

### Statistical analysis

We compared the baseline characteristics of the participants using analysis of variance tests (ANOVA) for continuous variables. Categorical variables were analysed using the chi-square test. Mean betatrophin and CVDs risk factors (LDL, HDL, and FBG) values were estimated within each group of homozygous referent (HR), heterozygous (HET), and homozygous variant (HV) genotypes for each SNP. The betatrophin, LDL, HDL, and FBG levels were adjusted by age, gender and BMI using analysis of covariance (ANCOVA) approach. Data is reported as mean ± standard deviation (SD) and range, unless stated otherwise. A *P*-value, 0.05 was considered to be statistically significant. All analyses were performed using SAS (version 9.4; SAS Institute, Cary, NC). Genotype frequencies were determined according to the Hardy-Weinberg equilibrium (HWE).

## Results

### Overall population characteristics and phenotype distribution

Our population was comprised of a total of 283 non-diabetic Arab subjects. Characteristics of the study population are outlined in Table 1. Using Sanger Sequencing method, two sequence variants were identified in ANGPTL8 gene (Rs.892066 and Rs.2278426) Fig. 1. A total of 283 Arab samples were sequenced, 188 (66.4 %) had homozygote form of Rs.892066 (C/C), while 85 (30.1 %) were heterozygotes (C/G) and only 10 (3.5 %) were homozygote (G/G) (Table 2). For the second sequence variant identified in ANGPTL8 Rs.2278426, a total of 248 (87.6 %) participants had homozygote form of this variant (C/C), while 35 (12.4 %) were heterozygotes (C/T), none of the sequenced samples had the homozygote (T/T) genotype (Table 3).

**Table 1:** Clinical and biochemical profile of the study population

Variables	Participants (*n* = 283)
Age (years)	41.39 ± 10.77
Male (%)	156 (55.8 %)
BMI (kg/m^2^)	31.49 ± 6.84
Waist/hip	0.90 ± 0.14
FBG (mmol/l)	5.00 ± 0.57
HBA1C (%)	5.37 ± 0.66
Insulin (mU/L)	9.02 ± 5.32
HOMAIR	2.04 ± 1.39
TRIG (mmol/l)	1.51 ± 0.80
HDL (mmol/l)	1.15 ± 0.37
LDL (mmol/l)	3.40 ± 1.00
Betatrophin ng/mL	808.82 (192.9–9305.56)
Rs.892066 (%)	
CC	188 (66.4 %)
CG	85 (30 %)
GG	10 (0.3.6 %)
Rs2278426 (%)	
(R59W)	
CC	248 (87.6 %)
CT	35 (12.4 %)

Results are reported as Mean ± SD except for non-normally distributed betatrophin that are presented as Median (range)

**Table 2:** Characteristics of the ANGPTL8 SNP Rs.892066 variants according to their genotype

Variable	SNP Rs.892066 (*N* = 283)			
	CC (*n* = 188)	CG (*n* = 85)	GG (*n* = 10)	*p*-value
Age	41.43 ± 0.79	41.24 ± 1.17	41.90 ± 3.42	0.9803
Gender				
Male	90 (31.8 %)	39 (13.8 %)	4 (1.4 %)	0.8621
Female	98 (34.6 %)	46 (16.3 %)	6 (2.1 %)	
BMI (kg/m^2^)	31.59 ± 0.49	30.92 ± 0.74	34.30 ± 2.16	0.3139
FBG (mmol/l)	4.98 ± 0.04	5.03 ± 0.06	5.03 ± 0.18	0.8391
HBA1C %	5.37 ± 0.05	5.35 ± 0.07	5.44 ± 0.21	0.9277
Insulin (mU/L)	9.71 ± .48	8.73 ± .72	11.56 ± 2.10	0.3196
HOMAIR	2.23 ± .13	2.0 ± .19	2.70 ± .55	0.3981
LDL (mmol/l)	3.41 ± 0.07	3.39 ± 0.11	3.50 ± 0.32	0.9752
HDL (mmol/l)	1.17 ± 0.03	1.11 ± 0.04	1.13 ± 0.12	0.4563
TCH (mmol/l)	5.19 ± 0.08	5.18 ± 0.12	5.31 ± 0.35	0.9382
TG (mmol/l)	1.47 ± 0.06	1.54 ± 0.09	1.94 ± 0.25	0.1715
Betatrophin ng/mL	791.7 (215.7–7264.3)	828.1 (192.9–9305.6)	815.0 (621.4–1310.8)	0.8600

Results are reported as Mean ± SE except for non-normally distributed betatrophin that are presented as Median (range)

**Table 3:** Characteristics of the ANGPTL8 SNP Rs.2278426 variants according to their genotype

Variable	Rs.2278426 (*N* = 283)		
	CC (*n* = 248)	CT (*n* = 35)	*p*-value
Age (Year)	41.53 ± 0.68	40.43 ± 1.82	0.5728
Gender			
Male	121 (42.8)	12 (4.2)	0.1075
Female	127 (44.9)	23 (8.1)	
BMI (kg/m^2^)	31.37 ± 0.44	32.29 ± 1.16	0.4622
FBG (mmol/l)	4.97 ± 0.04	5.19 ± 0.10	0.0372
HBA1C %	5.38 ± 0.04	5.30 ± 0.11	0.4751
Insulin (mU/L)	9.34 ± .42	10.49 ± 1.12	0.3353
HOMAIR	2.12 ± .11	2.49 ± .29	0.2381
LDL (mmol/l)	3.43 ± 0.06	3.23 ± 0.17	0.285
HDL (mmol/l)	1.16 ± 0.02	1.05 ± 0.06	0.1121
TCH (mmol/l)	5.21 ± 0.07	5.04 ± 0.18	0.3930
TG (mmol/l)	1.48 ± 0.05	1.71 ± 0.14	0.1030
Betatrophin ng/mL	812.5 (215.7–9305.6)	786.3 (192.9–1899.2)	0.5677

Results are reported as Mean ± SE except for non-normally distributed betatrophin that are presented as Median (range)

### Population characteristics stratified according to ANGPTL8 variants

**Fig. 2:**
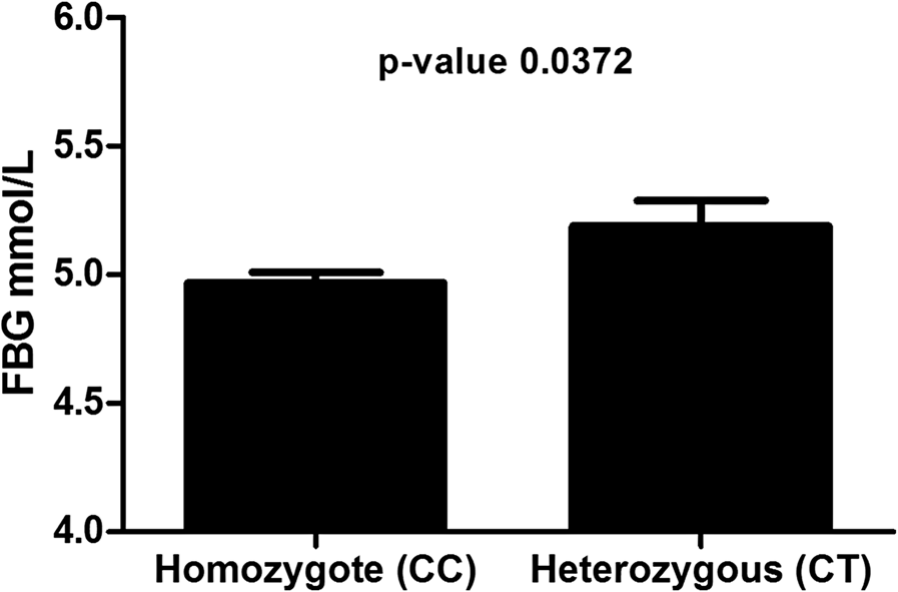
Fasting Blood Glucose level in the homozygote genotype and heterozygote form of the Rs.2278426 SNP. Heterozygote forms of the SNP showing higher level of FBG than the wild type. Adjusted for age, gender, and BMI

In order to identify differences between the two different genotypes for Rs.2278426 SNP, population was stratified according to sequence variant (wild type C/C or heterozygote C/T). Subjects with heterozygote form C/T had higher FBG level of (5.19 ± 0.10 mmol/L) than wild type C/C form (4.97 ± 0.04 mmol/L) (*p-*value = 0.00372) Fig. 2. No significant difference was observed in age, BMI and TC. Age for wild type was 41.53 ± 0.68 year and 40.43 ± 1.82 year for heterozygote (*p*-value =0.5728). BMI was 31.37 ± 0.44 for wild type and 32.29 ± 1.16 for heterozygote (*p*-value = 0.4622). No statistically significant difference was also observed in TC (wild type = 5.21 ± 0.07 mmol/L vs. 5.04 ± 0.18 mmol/L for heterozygote (*p*-value = 0.3930)).

### Lipid profile in different variants

**Fig. 3:**
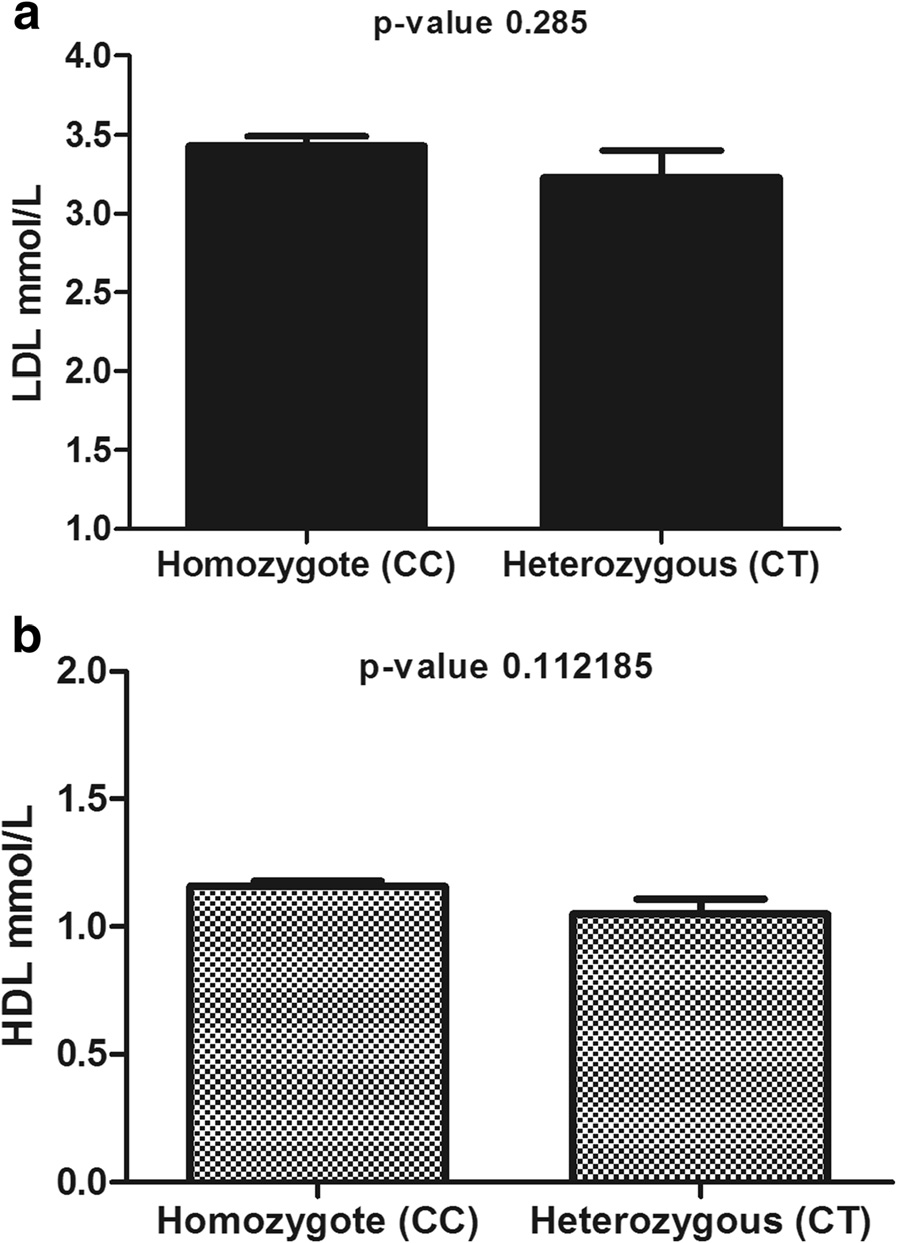
LDL and HDL level in the homozygote genotype and heterozygote form of the Rs.2278426 SNP. **a** LDL level in both homozygote and heterozygote forms of the ANGPTL8 Rs.2278426 variants. **b** HDL level in both homozygote and heterozygote forms of the ANGPTL8 Rs.2278426 variants. Adjusted for age, gender, and BMI

LDL and HDL levels showed a trend of decrease in the heterozygote form of Rs.2278426, which was not statistically significant. LDL level in the wild type form of ANGPTL8 was 3.43 ± 0.06 mmol/L compared to 3.23 ± 0.17 mmol/L (*p*-value = 0.1085) in the heterozygote form as seen in Fig. 3a. Similarly HDL also showed a similar trend of being lower in the heterozygote 1.05 ± 0.06 mmol/L vs. 1.16 ± 0.02 mmol/L in the wild type (*p*-value = 0.1121) Fig. 3b. Finally, circulation level of ANGPTL8 did not show significant difference between both forms, wild type was 812.5 ng/mL (215.7–9305.6) vs. 786.3 ng/mL (192.9–1899.2) for the heterozygote (*p*-value = 0.5677). No significant difference in FBG, LDL, HDL, TG or betatrophin level was observed between the different variants of Rs.892066.

## Discussion

ANGPTL8 is a recently identified protein that has been shown to play a role in lipid metabolism. Sequence variants of ANGPTL8 gene has been associated with decreased LDL and HDL level in African Americans and Hispanic [12]. This study was aimed at identifying genetic difference in the sequence of ANGPTL8 gene in Arabs and exploring their association with metabolic risk factors. Our data showed for the first time that Arab subjects with the C/T heterozygote genotype of Rs.2278426 variant had higher FBG level compared to the wild type. No difference in FBG was observed between other genetic variants. On the other hand, subjects with the C/T genotype of Rs.2278426 did not show significant difference in their LDL and HDL level compared to homozygote genotype. The trend however, was not statistically significant. Circulation level of ANGPTL8 was not affected by the various genotypes for both SNPs.

ANGPTL8 is a recently characterized protein that is mainly produced in the liver and adipose tissue. It has been established as a regulator of lipid metabolism. A sequence variant in betatrophin (Rs2278426, c.194C > T; R59W) has been shown to be associated with lower plasma LDL-C and HDL-C [12]. Ethnic variation has been associated with variable minor allele frequency (MAF) rates for this variant in respect to LDL-C and HDL-C plasma levels [12]. Hispanic had the highest 59 W MAF of 26 %, followed by African American (18 %) and the least was reported in European Americans (5 %) [12]. This variant was associated with lower LDL-C and HDL-C in African Americans and Hispanic but not European Americans in the Dallas Heart Study [12]. No association was observed with TG level in any of the ethnicities [12]. In this study and in line with earlier reports, we showed a trend towards lower LDL and HDL level in the R59W variant, however, it was not statistically significant F.

In this study we showed that Arabs with the CT heterozygote form of Rs.2278426 had a higher level of FBG compared to those with CC homozygote form. This data suggest that ANGPTL8 plays a role in glucose metabolism in Arabs. ANGPTL8 or betatrophin role in beta-cell proliferation has been recently questioned. Gusarova et al. has suggested that betatrophin was not responsible for any beta-cell proliferation and mice lacking ANGPTL8 had normal glucose metabolism under insulin resistance conditions [12]. Similar data was observed by Quagliarini et al. in Hispanic participants of the Dallas Heart Study (DHS) study but not in African Americans or Europeans [12]. In the DHS cohort, Hispanic subjects with the homozygote form had significantly higher FBG (100 ± 9 mg/dL) compared to homozygote (CC) genotype that had (93 ± 11 mg/dL) [12]. However, this was only observed in a very small number of participants which was ignored in their discussion. Even though the exact mechanism for the effect of this sequence variation has not been well studied, it’s possible to speculate that the betatrophin protein structure will be affected due to the change in the charge of the amino acid residue from R to W. A similar R to W mutation has been previously reported and was shown to disrupt functional domains within Troponin T or other proteins [25, 26]. The functional impact of this mutation on the function of ANGPTL8 requires further investigation. Our data further highlight the importance of ethnicity in understanding the role of ANGPTL8 and its sequence variants in its cellular function. These data combined still suggest a role for ANGPTL8 in glucose metabolism that may not involve beta-cell proliferation. It will also require further functional analysis to better understand this functional difference and perhaps the cross-talk with other ethnically specific determinants.

Prospective studies on determining causality and the involvement of ANGPTL8 in development of T2D is still lacking and constitutes one of the limitations of the current study as it represents a cross sectional study. Another limitation of the current study is the sample size. Perhaps the lack of statically significant association between R59W variations and LDL and HDL is caused by this relatively small sample size. The other factor for the different trend is the ethnic variation of the current population where we only studied Arabs. This is due to the use of Sanger sequencing to sequence the whole gene instead of looking at a specific SNP. Nonetheless, one of the main strength of this study is that it highlights the importance of the ANGPTL8 gene in a high risk population that is not very well studied.

## Conclusions

In this study we showed that ANGPTL8 R59W gene variant is associated with higher FBG level in non-diabetic Arabs compared to the wild type. 59 W showed a trend toward lower LDL and HDL levels, similar to African Americans and Hispanic population, however without being statistically significant. On the other hand, ANGPTL8 level in plasma was not affected by either SNP. In conclusion, role of ANGPTL8 in glucose metabolism requires further investigation and ethnicity seems to be a major determinant of the function of ANGPTL8 and better understanding of ethnic differences will be required to establish the functional role of betatrophin in multiple ethnicities.
